# Stroke caused by an inflammatory thrombus: a case report

**DOI:** 10.1186/s12883-017-0816-3

**Published:** 2017-02-16

**Authors:** Kai Shan, Wei Guo

**Affiliations:** Emergency Department, Beijing Tiantan Hospital, Capital Medical University, Beijing, 100050 China

**Keywords:** Stroke, Infection, *Staphylococcus aureus*, Inflammatory thrombus, Haemorrhagic transformation

## Abstract

**Background:**

Stroke is the leading cause of mortality and disability worldwide. Several definite risk factors have been identified for stroke, although infectious factors might also contribute to stroke episodes through increased susceptibility or direct induction.

**Case presentation:**

A 46-year-old Chinese male initially presented with fever, headache, and impaired memory and developed disturbance of consciousness after admission. A clinical diagnosis of *Staphylococcus aureus* sepsis, massive cerebral infarction and haemorrhagic transformation (left internal carotid arterial system, inflammatory thrombus) were made based on brain radiography, blood culture and postoperative pathological examinations. These symptoms improved following antibiotic therapy with vancomycin and conventional treatments for stroke.

**Conclusion:**

For stroke patients without traditional cerebrovascular risk factors but with signs of infection, infectious causes should be considered.

## Background

Stroke is the leading cause of mortality and disability worldwide [1], and several definite risk factors have been identified for stroke. As shown by the INTERSTROKE study, approximately 90% of stroke episodes are associated with hypertension, smoking, heart disease, diabetes and six additional risk factors [2]. However, infectious factors might also contribute to stroke episodes through increased susceptibility or direct induction [3]. In this paper, one case admitted to our hospital due to stroke induced by *Staphylococcus aureus* sepsis is described with the purpose of increasing clinicians’ understanding of infectious stroke.

## Case presentation

**Fig. 1:**
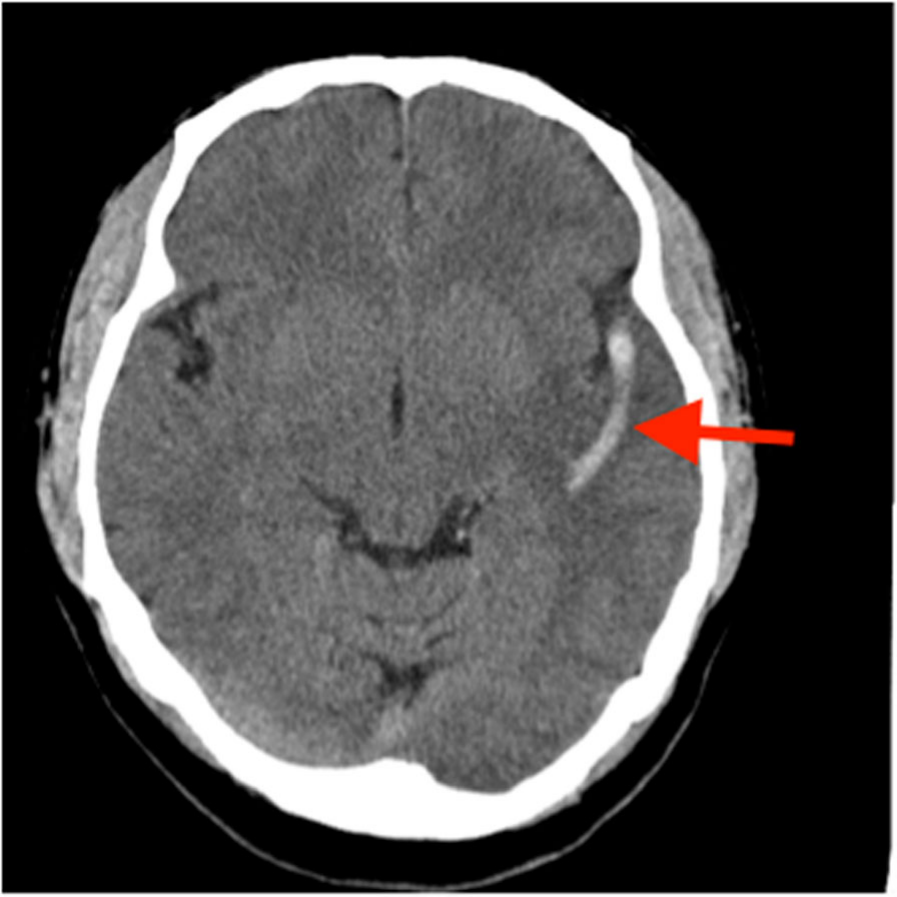
A brain computed tomography image showing the thrombus in the left middle cerebral artery (arrow)

A 46-year-old male visited the emergency department of our hospital on April 23, 2015 due to fever and headache for three days and impaired memory for half a day. The patient had experienced fever three days before, with a peak body temperature of 38.5 °C. No other discomfort was described. He had visited other hospitals before and was diagnosed with an upper respiratory infection based on haematological findings. However, his condition did not improve after treatment with intravenous levofloxacin. Within a half a day, he developed impaired memory, concomitant with speech disturbance, poor response, no significant limb movements and convulsions before being referred to the emergency department of our hospital. The patient had no medical history. No histories of food and drug allergies were obtained. The patient denied any prior history of smoking, heavy drinking or drug abuse. On arrival, the initial physical examination revealed the following: body temperature 38.0 °C, pulse 100 beats/min, respirations 26 breaths/min, blood pressure 144/76 mmHg, delirium, speech disturbance, equal size and round shape of bilateral pupils with diameters of approximately 3.0 mm, sensitive to light reflex, basically normal cranial nerve examination, limb muscle strength Grade V, negative bilateral pathological signs, and no ataxia. The patient’s neck was soft, and there were no signs of meningeal irritation. No significant findings were found on the cardiopulmonary and abdominal examinations. No skin rashes or bleeding sites were observed after examining the entire body. The laboratory test results were the following: haematology: white blood cells (WBC), 12.5 × 10^9^/L, neutrophil percentage, 87.6%; C-reactive protein (CRP), 56 mg/L; procalcitonin (PCT), 2.8 ng/mL; and D-dimer, 3.2 μg/mL. A chemistry panel and screens for infectious diseases and cardiac markers were generally normal. No abnormal findings were observed on chest X-ray or electrocardiogram. Brain computed tomography (CT) showed a curved high-density mass at the left sylvian fissure area and an ischemic lesion of the left temporal lobe (Fig. 1).

As an emergency diagnosis, massive cerebral infarction (probable atherosclerosis of the left internal carotid artery system) was considered, and intracranial infection could not be excluded; appropriate treatments included aspirin for anti-platelet aggregation, atorvastatin for lipid regulation and plaque stabilization, and cefuroxime and acyclovir for anti-infection.

**Fig. 2:**
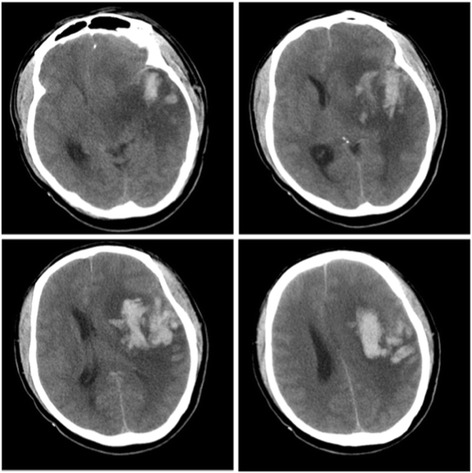
Brain computed tomography images showing left temporal lobe insular lobe infarction and haemorrhagic transformation

The patient experienced aggravated unconsciousness after two hours of admission, and physical examination showed the following: lethargy, aphasia, equal size and round shape of bilateral pupils with diameters of approximately 3.0 mm, sensitive to light reflex, shallow right nasolabial folds, immobilization of right limbs upon orbital pressure, movable left limbs, positive right pathological signs and negative left pathological signs. Further revascularization therapy was considered, and the patient was immediately transferred to undergo brain magnetic resonance imaging (MRI). On the way to the radiology department, the patient experienced further aggravation of unconsciousness and entered a light coma, concomitant with limb seizure, gazing to the left of bilateral eyes, equal size and round shape of bilateral pupils with diameters of approximately 2.0 mm, and disappearance of the light reflex. Appropriate treatments included midazolam for epilepsy, mannitol for dehydration and intracranial hypotension. After termination of the seizure, re-examination of the brain CT showed a curved high-density mass at the left sylvian fissure area and a massive low-density area in the left temporal lobe with many high-density plot areas scattered within it. CT images also showed compressed deformation of the left ventricle and a shift of the brain midline to the right (Fig. 2). Considering that the patient developed haemorrhagic transformation, he was admitted to the emergency intensive care unit for further management.

**Fig. 3:**
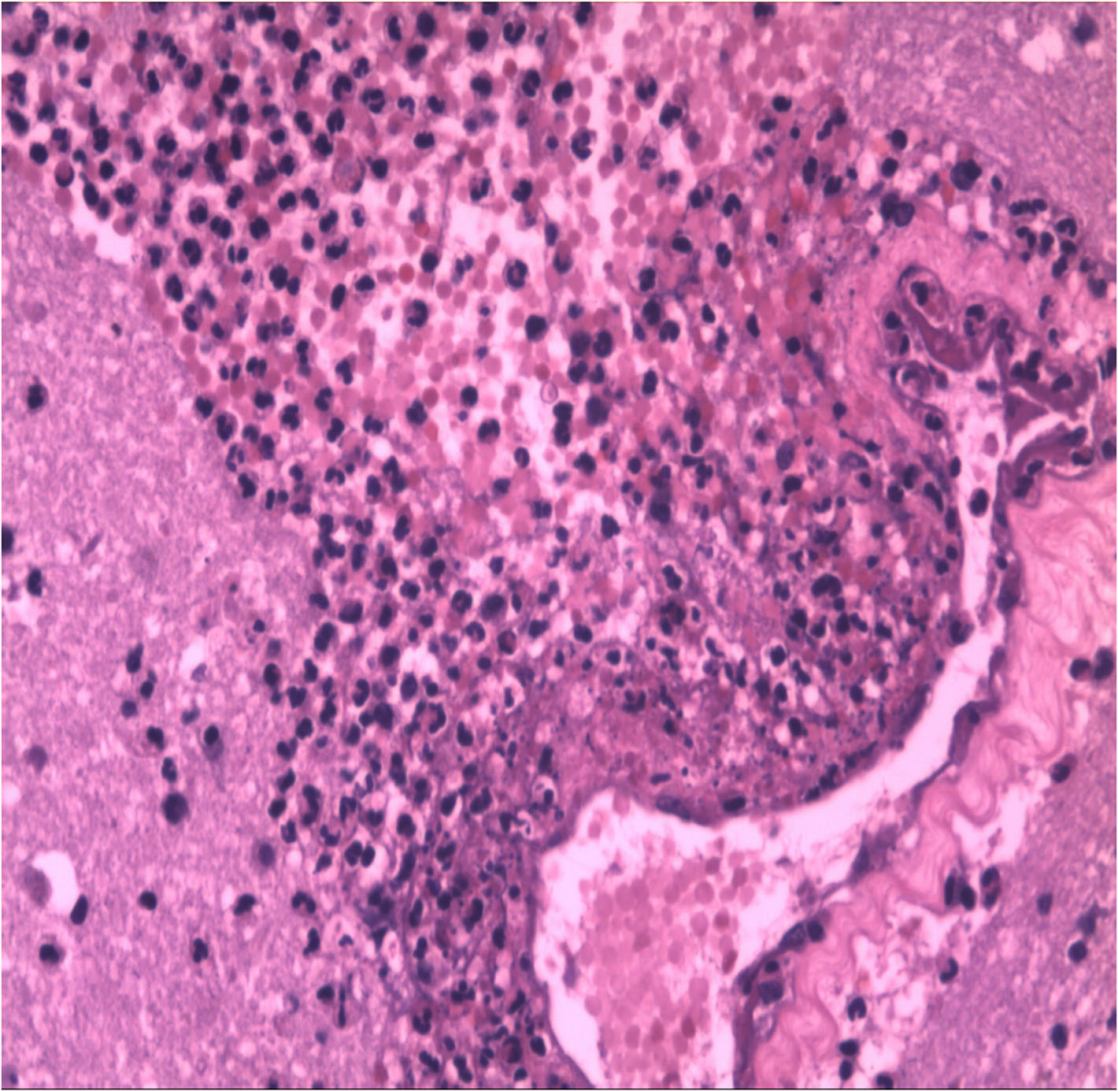
Postoperative histopathological examination showing vasculitis and perivascular inflammatory exudates (hematoxylin-eosin staining, ×200)

**Fig. 4:**
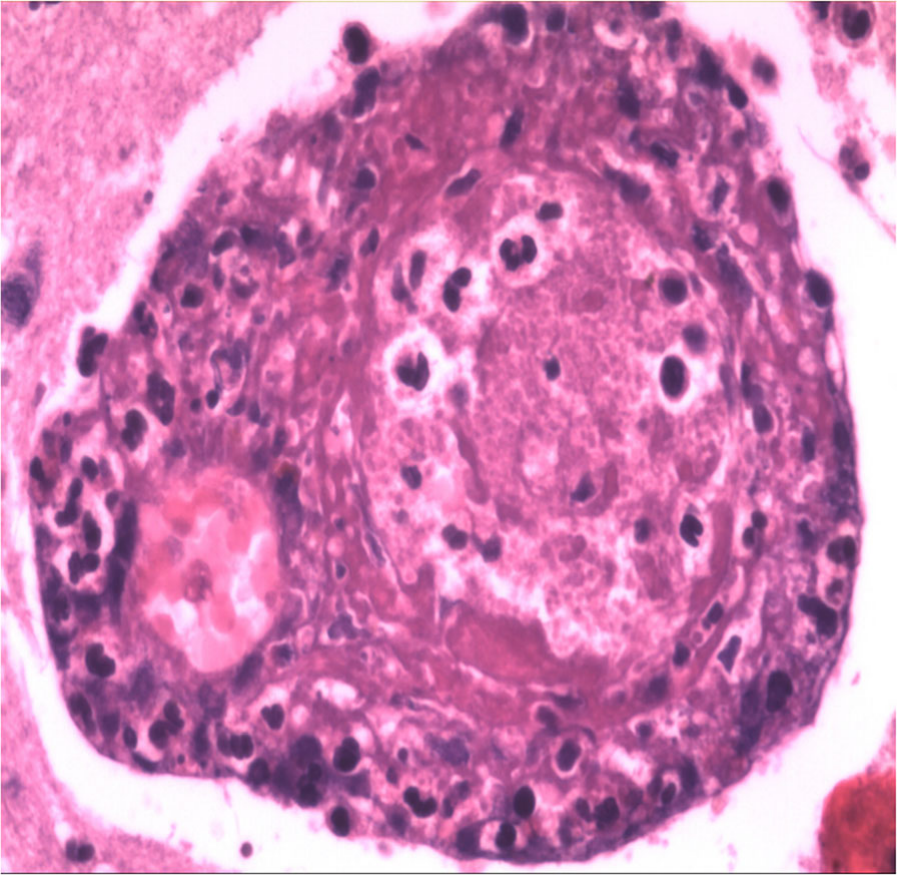
Postoperative pathological examination showing the inflammatory thrombus in the artery (hematoxylin-eosin staining, ×400)

Eighteen hours after admission, the patient experienced further aggravation of unconsciousness and entered a moderate coma, concomitant with high fever (body temperature 39.9 °C), bilateral pupils: left versus right = 5.0 mm versus 3.0 mm, respectively, disappearance of the light reflex, increased muscle tone of the right limbs, and positive pathological signs of bilateral limbs. The brain CT re-examination showed expansion of the bleeding area, with overt signs of brain herniation. The patient underwent emergency left frontal temporal craniotomy, removal of the intracranial hematoma and a decompressed craniotomy performed by neurosurgeons. The intraoperative findings were as follows: dark red appearance of the left temporal brain tissue, concomitant with subarachnoid haemorrhage (SAH); an ostomy of approximately 0.2 cm was created along the left temporal sulcus; a visible purple blood clot was observed, concomitant with surrounding contused brain tissues; and a large vascular embolization could be observed in the hematoma cavity. Approximately 10 mL of the hematoma and contused brain tissues were removed and sampled for pathological evaluation. Histopathological examination showed haemorrhage, oedema and inflammatory changes in brain tissues, neutrophil infiltration into the vascular walls and surrounding tissues, and microvascular purulent thrombosis (Figs. 3–4). The patient was transferred to the neurologic intensive care unit for further treatment after the operation. During the course of the treatment, negative findings were found for *Chlamydia pneumoniae*, mycopsslasma, influenza, tuberculosis, the TORCH panel, hepatitis B, hepatitis C, syphilis and human immunodeficiency virus (HIV); the sputum culture was negative, and the blood culture suggested *Staphylococcus aureus*. No abnormal findings were found on chest X-ray or ultrasound. In addition, two transthoracic echocardiography (TTE) examinations were normal, and there were no observations of valvular dysfunction or neoplasm. The following final diagnosis was considered: massive cerebral infarction, consistent with haemorrhagic transformation (the left internal carotid artery system, inflammatory thrombus), *Staphylococcus aureus* sepsis and symptomatic epilepsy. After two months of vancomycin anti-infective treatments and supportive and symptomatic treatment, the consciousness of the patient improved gradually, and several blood cultures were negative. He underwent rehabilitation and was discharged from the hospital on the 72^nd^ postoperative day (POD). At discharge, his Glasgow Coma Scale (GCS) was E4V4M6, and the modified Rankin scale score was 3.

## Discussion

The patient described herein was middle-aged and healthy; although no stroke risk factors were identified, a rapid onset of disease was observed. After admission, the brain CT suggested cerebral infarction, subsequently followed by haemorrhagic transformation. After craniotomy, the pathological examination indicated that the cerebral infarction was induced by inflammatory thrombosis. Additionally, the patient also experienced fever, shortness of breath and unconsciousness. Laboratory examinations suggested abnormal elevations of haematological variables, CRP, PCT and D-dimer. *Staphylococcus aureus* was indicated by blood culture. This condition responded well to vancomycin treatment. Therefore, the sepsis was considered to be induced by *Staphylococcus aureus* infection.

Although there were definite risk factors for stroke, all episodes of this disease could not be fully explained by the factors currently identified. Indeed, many patients who develop stroke do not have any risk factors, and no definite aetiologies are identified for approximately one-third of stroke cases. As shown by several studies, there might be a correlation between systemic infection and episodes of stroke [4]. Indeed, relevant case–control studies have confirmed this correlation between systemic infection and stroke. In patients with stroke, the odds ratio of a precursor infection was in the range of 2–14.5 [5–10].

A wide range of pathogens has been found to be associated with an elevation of stroke risk, including viruses, bacteria, fungi and parasites. The most investigated pathogens include *Helicobacter pylori*, *Chlamydia pneumoniae*, *Mycoplasma pneumoniae*, *Haemophilus influenzae*, Epstein-Barr virus (EBV), herpes simplex virus (HSV)-1, HSV-2 and cytomegalovirus [3, 11].

*Staphylococcus aureus* is a major human pathogen that causes a wide range of clinical infections [12]. *Staphylococcus aureus* bacteraemia (SAB) is associated with a short-term increased risk of stroke, and the risk can persist for up to 180 days. The risk factors for stroke after SAB include old age, prior arterial thromboembolic events, atrial flutter/fibrillation, hypertension and endocarditis [13].

The mechanism of stroke induced by infection remains incompletely understood, although stimulation of the inflammatory response is considered to be the main driver for stroke [14–16]. Pathogens may directly invade the vascular wall during the course of infection, concomitant with an increase in smooth muscle cell proliferation or inflammatory cytokine production. Additionally, regions far away from the primary infected loci might also be affected, and this secondary impairment might also lead to damage of the arterial wall. Furthermore, the inflammatory response induced by infection could stimulate enhancement of platelet aggregation and dysfunction of vasodilation [15]. It has been described that blood levels of high-sensitivity CRP (HS-CRP) may be considered an independent predictor for ischemic stroke, although the exact correlation remains unclear [17, 18].

Based on the current patient’s medical history, physical examination, and laboratory, radiology and pathological examinations, the aetiology of stroke was considered to be embolic stroke and subsequent haemorrhagic transformation as a result of migration of the septic emboli induced by systematic *Staphylococcus aureus* infection to the left internal carotid artery system. *Staphylococcus aureus* is a common pathogen of acute infective endocarditis, and infective endocarditis has been identified as an important cause of cardioembolic stroke [19–21]. However, two TTE examinations showed no abnormal findings. A diagnosis of infective endocarditis was still possible for this case because one major and one minor criterion of the modified Duke criteria were met [22]. Further cardiac evaluation, such as a transoesophageal echocardiography (TEE), should have been considered. Unfortunately, due to the serious condition of the patient and the unavailability of bedside TEE in our hospital, TEE was not performed in this case.

Additionally, post-operational pathological findings suggested inflammatory changes of brain tissues, consistent with purulent thrombosis. However, a culture of brain tissue was not obtained because of the poor communication between the emergency physicians and neurosurgeons during such an urgent situation. If the same strain were identified between this culture and the blood culture, the aetiological diagnosis of this case would be further confirmed.

## Conclusion

Appropriate aetiological judgment is critical for the treatment of stroke. In clinical practice, the possibility of stroke induced by infective factors should be considered with caution for patients with no traditional risk factors of stroke and infective signs. For these patients, an appropriate regimen should be determined based on targeted anti-infective therapies in addition to traditional treatments for stroke. Additionally, similar to traditional risk factors, preventive interventions against some infections might also contribute to decreasing the incidence of stroke in some cases.

## Abbreviations

CRP: C-reactive protein
CT: Computed tomography
EBV: Epstein-Barr virus
GCS: Glasgow coma scale
HIV: Human immunodeficiency virus
HS-CRP: High-sensitivity C-reactive protein
HSV: Herpes simplex virus
MRI: Magnetic resonance imaging
PCT: Procalcitonin
POD: Postoperative day
SAB: *Staphylococcus aureus* bacteraemia
SAH: Subarachnoid haemorrhage
TORCH: Toxoplasmosis other infections, rubella, cytomegalovirus, and herpes simplex virus
WBC: White blood cell
